# Current challenges in distinguishing climatic and anthropogenic contributions to alpine grassland variation on the Tibetan Plateau

**DOI:** 10.1002/ece3.4099

**Published:** 2018-04-27

**Authors:** Lanhui Li, Yili Zhang, Linshan Liu, Jianshuang Wu, Shicheng Li, Haiyan Zhang, Binghua Zhang, Mingjun Ding, Zhaofeng Wang, Basanta Paudel

**Affiliations:** ^1^ Key Laboratory of Land Surface Pattern and Simulation Institute of Geographic Sciences and Natural Resources Research CAS Beijing China; ^2^ University of Chinese Academy of Sciences Beijing China; ^3^ CAS Center for Excellence in Tibetan Plateau Earth Sciences Beijing China; ^4^ Freie Universität Berlin Institute of Biology Biodiversity/Theoretical Ecology Berlin Germany; ^5^ School of Public Administration China University of Geosciences Wuhan China; ^6^ Jiangxi Normal University Nanchang China

**Keywords:** alpine grassland, climate change, degradation, human activity intensity, Tibetan Plateau, validation

## Abstract

Quantifying the impact of climate change and human activities on grassland dynamics is an essential step for developing sustainable grassland ecosystem management strategies. However, the direction and magnitude of climate change and human activities in driving alpine grassland dynamic over the Tibetan Plateau remain under debates. Here, we systematically reviewed the relevant studies on the methods, main conclusions, and causes for the inconsistency in distinguishing the respective contribution of climatic and anthropogenic forces to alpine grassland dynamic. Both manipulative experiments and traditional statistical analysis show that climate warming increase biomass in alpine meadows and decrease in alpine steppes, while both alpine steppes and meadows benefit from an increase in precipitation or soil moisture. Overgrazing is a major factor for the degradation of alpine grassland in local areas with high level of human activity intensity. However, across the entire Tibetan Plateau and its subregions, four views characterize the remaining controversies: alpine grassland changes are primarily due to (1) climatic force, (2) nonclimatic force, (3) combination of anthropogenic and climatic force, or (4) alternation of anthropogenic and climatic force. Furthermore, these views also show spatial inconsistencies. Differences on the source and quality of remote sensing products, the structure and parameter of models, and overlooking the spatiotemporal heterogeneity of human activity intensity contribute to current disagreements. In this review, we highlight the necessity for taking the spatiotemporal heterogeneity of human activity intensity into account in the models of attribution assessment, and the importance for accurate validation of climatic and anthropogenic contribution to alpine grassland variation at multiple scales for future studies.

## 1 INTRODUCTION

Climate change and human activities are the two primary driving factors for changes in global ecosystems (Haberl et al., 2007; Vitousek, Mooney, Lubchenco, & Melillo, 1997). Climate change, especially global warming, has greatly affected ecosystems ranging from polar terrestrial to tropical marine (Walther et al., 2002). Due to the high spatial heterogeneity in climate change in terms of magnitude and direction (IPCC, 2014), ecosystem dynamics induced by climate change varied among different regions, especially in fragile and sensitive ecosystems at high altitude or latitude (Seddon, Macias‐Fauria, Long, Benz, & Willis, 2016; Zhu et al., 2016). Meanwhile, anthropogenic global environmental change has facilitated the Earth entering a human‐dominated geological era termed the Anthropocene (Lewis & Maslin, 2015). Nearly, three‐quarters of the snow‐uncovered land surface have experienced measurable human pressures (Venter et al., 2016a, 2016b), which affect the interaction between land surface and regional climate (Pitman et al., 2011) and ecosystem structure and function (Bateman et al., 2013; Turner, Lambin, & Reenberg, 2007). A growing number of scientists are focused to distinguish the respective contribution of climatic and anthropogenic forces to ecosystem dynamics, which is critical for a more sustainable ecosystem management in the future (Haberl, Erb, & Krausmann, 2014; Rockstrom et al., 2009; Wang et al., 2011).

**Figure 1:**
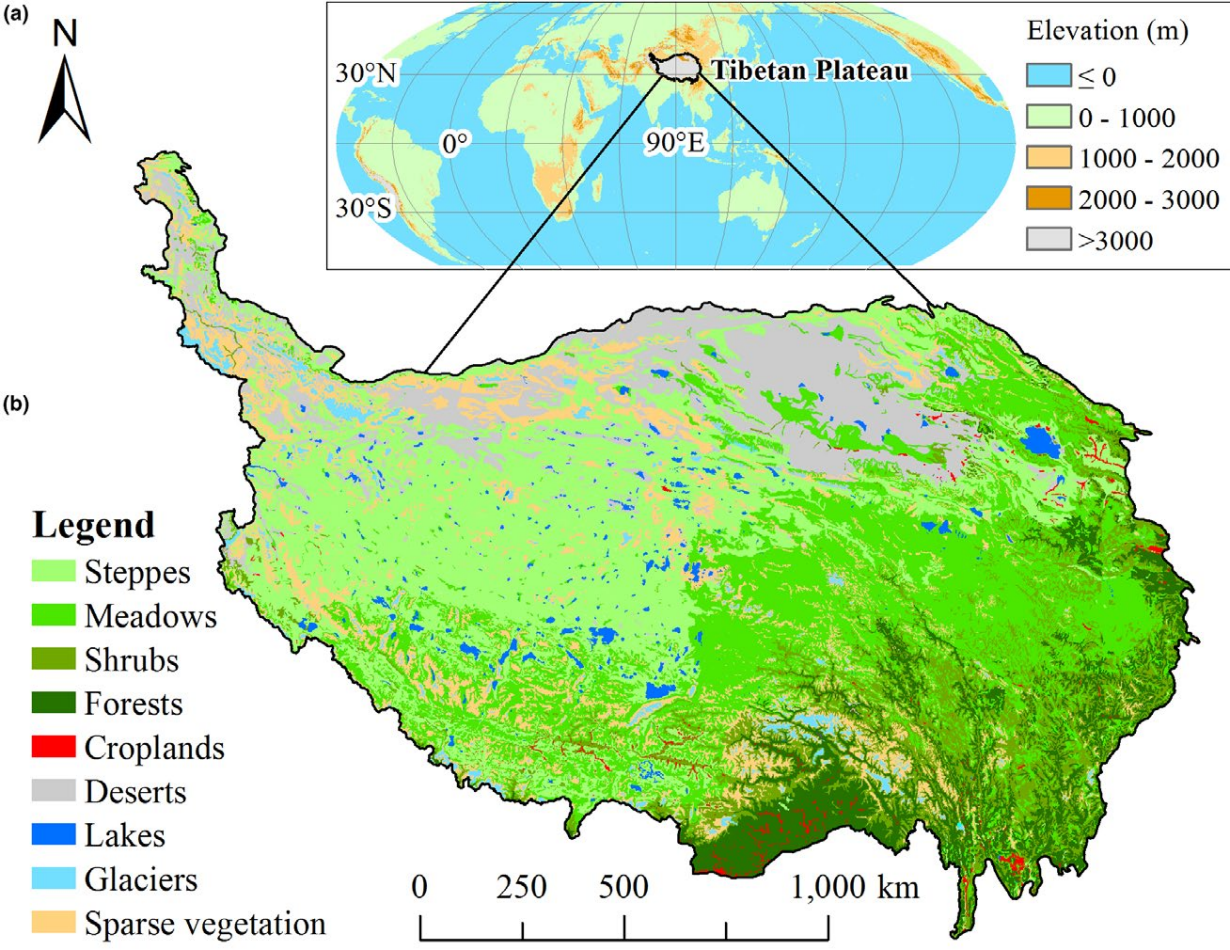
Spatial distribution of topographic feature (a) and typical vegetation types (b) on the Tibetan Plateau

The Tibetan Plateau is known as “roof of the world” and “third pole of the Earth” (Figure 1) (Qiu, 2008; Zhang, Li, & Zheng, 2014, 2014), where alpine ecosystems are as vulnerable and sensitivity as those in the Arctic and Antarctic regions to climate change and human activities (Chen et al., 2013; Li, 2017; Yao et al., 2016). During the past several decades, Tibetan Plateau has experienced a more significant warming than other places on the earth (Pepin et al., 2015; Yang et al., 2014) and a slight increase in precipitation with apparent spatial heterogeneity (Kuang & Jiao, 2016). Although human activity intensity is low overall on the Tibetan Plateau, its variation is greater than that of the whole world over past two decades (Li, Zhang, Wang, & Li, 2017). Therefore, the Tibetan Plateau is an ideal location to investigate the response of vegetation growth to climate change and human activities (Piao, Fang, & He, 2006; Xu, Wang, & Zhang, 2016). Alpine grassland, including alpine meadow and alpine steppe, is the main vegetation type on the Tibetan Plateau and covers two‐thirds of land surface on this plateau (Figure 1). Due to the dual effects of climatic change and human activities, alpine grassland in local areas has shown different extents of degradation over the past decades (Harris, 2010; Li et al., 2013; Wang, Lassoie, Morreale, & Dong, 2015). This could affect livehood for local people (Fan, Xu, Wang, & Niu, 2015), biological geochemistry circulation (Chen et al., 2013; Liu, Zamanian, Schleuss, Zarebanadkouki, & Kuzyakov, 2018), ecosystem services (Ouyang et al., 2016), and even threaten the ecological security of China and South Asia (Sun, Zheng, Tandong, & Zhang, 2012). However, due to ecological construction and environmental conservation projects conducted by the government, the overall health of the alpine grassland on Tibetan Plateau has improved, except for some local areas becoming degraded (Zhang, Qi, et al., 2014).

Presently, an increasing number of researchers are paying attention to quantitatively assess the relative influence of climate change and human activities on alpine grassland dynamic across Tibetan Plateau. However, uncertainties even controversies exist among the conclusions of such studies. Distinguishing the respective impact of climate change and human activities on alpine grassland dynamic remains challenges (Feng, Wu, Zhang, Zhang, & Song, 2017; Wang, Zhang, et al., 2016; Zhang, Yang, et al., 2015), and till date no study has comprehensively reviewed these challenges. In this study, we review the methods for identifying the driving factors in variations of alpine grassland productivity at various scales, and the main conclusions. We also discuss the current disagreements and potential causes. Finally, we propose a potential roadway in order to face the recent challenges.

## 2 OVERVIEW OF THE METHODS FOR IDENTIFYING THE DRIVING FACTORS

The methods for identifying the contribution of climatic and anthropogenic forces to alpine grassland changes across Tibetan Plateau could be characterized as three types: manipulative experiments, traditional statistical analyses, and modeling with remote sensing datasets.

### 2.1 Manipulative experiments

Researchers generally control several climatic factors or human activity intensity in manipulative experiments. Warming and grazing experiments are the two primary types of manipulative experiments. Open‐top chambers (OTCs) (Ganjurjav et al., 2016) and infrared heaters (Ma et al., 2017; Wang et al., 2012) were set up to simulate climate warming. In field experiments that temperature and moisture were controlled, the reactions of vegetation growth were detected under different conditions, the relative impact of changing temperature or moisture on was quantified. In grazing‐manipulative experiments, researchers investigated vegetation traits, for example, structure, biomass, and diversity under different grazing regimes, such as permanent grazing, seasonal grazing, and grazing exclusion (Sun et al., 2014), or under different grazing pressures, from no grazing (fencing), moderate grazing intensity, to overgrazing (Li, Cao, et al., 2017; Miao, Guo, Xue, Wang, & Shen, 2015; Wang & Wesche, 2016). In grazing experiment studies, temperature and precipitation observed from meteorological stations in the grazing experimental areas were used to detect the complex interplay between different grazing regimes and climate change.

Warming experiments are usually conducted under no grazing conditions, while grazing experiments are conducted considering the impact of both human activities and climate change. In addition, to quantify the impact of climate change and grazing intensity on qualities and biodiversity of alpine grassland, controlled warming–grazing experiments have also been designed (Klein, Harte, & Zhao, 2007; Wang et al., 2012).

### 2.2 Traditional statistical analysis

Based on long‐term socioeconomic statistics, climate records, and remote sensing products, traditional statistical analysis has been widely carried out to quantitatively assess the influence of climatic and anthropogenic factors on alpine grassland change. Correlation analysis and partial correlation analysis were usually used to explore the relationship between climatic factors and the Normalized Difference Vegetation Index (NDVI) or net primary productivity (NPP) (Cong et al., 2017; Ding et al., 2007; Shen et al., 2016). Analytic hierarchy process (AHP) and principal component analysis (PCA) could provide acceptable results for quantifying the influence of climate change and human disturbance at a fine scale (Li et al., 2010; Zhou et al., 2005). However, anthropogenic data that were primarily derived from statistical data cannot well describe the spatially heterogeneous influence of human activities on alpine grassland. According to the distance attenuation theory, buffer analysis always was built to simulate the decreasing human activities with increasing distance from infrastructure, such as settlements and roads (Wang & Wesche, 2016). Once a relationship between accessibility factor and vegetation indices has been established, the method could assess the influence of human activities on alpine grassland (Liu et al., 2006; Zhao et al., 2015). However, in this method, a part of human influence may be underestimated in areas where it is not near settlements but belong to pastures.

### 2.3 Residuals‐Trend model

At a regional scale, the Residuals‐Trend model is the most widely applied to quantitatively assess the influence of climate and anthropogenic factors on alpine grassland over the Tibetan Plateau (Cai, Yang, & Xu, 2015; Chen et al., 2014; Wessels, Prince, Frost, & van Zyl, 2004). This method is based on the hypothesis that potential vegetation growth is only controlled by climate change. Thus, the human‐induced vegetation change could be detected after removing the impact of climate change (Wessels et al., 2004, 2007). NDVI and NPP are the most widely used indicators to monitor vegetation characteristics in studies using this method.

For NDVI as the representative index, this method is referred to as the NDVI‐based residual trend (RESTREND) (Evans & Geerken, 2004). Based on the ideal statistical relationship between annual peak NDVI time series and relevant climatic factors, such as precipitation and temperature (Cai et al., 2015), the NDVI change controlled by climate change can be predicted. Subsequently, the vegetation variation caused by human activities could be calculated, that is, the residuals between the predicted NDVI values and observed NDVI values. Finally, the trend of residuals could be calculated. A positive trend indicates that vegetation restoration is primarily driven by human activities, and vice versa. The reliability of result estimated by this method is highly dependent on the linear relationship between the variations in the vegetation index and climate factors (Wessels, van den Bergh, & Scholes, 2012).

When using NPP as the representative index, actual NPP (NPP_A_) is first simulated using remote sensing retrieval model, such as the Carnegie–Ames–Stanford Approach (CASA) model (Potter et al., 1993), and then, the potential or climatic NPP (NPP_P_) is simulated with climate model, such as the terrestrial ecosystem model (TEM) (Raich et al., 1991), and Thornthwaite Memorial model (Li, Zhang, Shen, Jia, & Li, 2016). Thus, the human‐induced NPP (NPP_H_) is defined as the residual between potential and actual NPP (NPP_H_ = NPP_P_ − NPP_A_), which represents the anthropogenic impact on variations in alpine grassland. Finally, the relative contribution of climatic and anthropogenic factors to alpine grassland changes can be identified by comparing the trends of NPP_H_ and NPP_P_ (Chen et al., 2014). Besides, this situation that both climate change and human activities were not the leading factor in alpine grassland change was not analyzed in recent study cases (Wang, Zhang, et al., 2016).

Generally, the methods of attribution assessment mentioned above vary with the study scales. Specifically, manipulative experiments were usually designed and carried out at local areas with one or several typical vegetation types (Ma et al., 2017; Wang et al., 2012). In the studies with buffer analysis, areas with intense variation in human activity intensity, such as degraded grassland in the Three Rivers Headwaters Region (Liu et al., 2006), are often selected. For assessments with Residuals‐Trend model, studies were performed at regional or macro scales, with different land cover types, climate patterns and human activity intensities covered. However, manipulative experiments primarily focused on the mechanisms of vegetation growth, while macro‐scale studies generally focused on calculating the proportions or areas of alpine grassland variation. Compared with the latter type, the studies based on manipulative experiments more exhaustively investigate the attributions of vegetation variation. Thus, over certain time periods and spatial locations, results based on experiments and field observations in a typical vegetation area could be used to validate and elucidate the results from larger scale studies based on related models.

## 3 ATTRIBUTION RESULTS

### 3.1 The impact of climate change on alpine grassland

According to field observations and manipulative experiments, temperature and soil moisture collectively affect plant growth in alpine grassland (Ma et al., 2017; Wu, Wurst, & Zhang, 2016). The responses of aboveground biomass to warming depend on vegetation types. The height of grasses in alpine meadows increases with increasing temperature (Ganjurjav et al., 2016), resulting an increase in aboveground biomass (Li, Wang, Yang, Yongheng, & Guangsheng, 2011; Wang et al., 2012). However, biomass in alpine steppes might decrease with increasing temperature (Ganjurjav et al., 2016), because a dramatic reduction in soil moisture could trigger severe droughts (Li et al., 2011; Wang et al., 2012). In addition, the impact of increasing temperature on vegetation coverage varies with different plant species (Zhang, Gao, et al., 2015). For instance, climate warming would increase coverage of forb, while decrease coverage of graminoids and legumes (Wang et al., 2012) and the percentage of palatable plants (Klein et al., 2007).

The spatially heterogeneous temporal trends in temperature and precipitation appeared on the Tibetan Plateau both over the past decades (Figure S1) (Kuang, Liu, Dong, Chi, & Zhang, 2016; Yang et al., 2014), which might induce complex responses in vegetation dynamics (Cong et al., 2017; Zhang, Yang, et al., 2015). Based on long‐term records of vegetation indices from satellite constellations, several studies show that warming would advance the green‐up date, delay the withering period, and lengthen the growing season on the Tibetan Plateau (Ding et al., 2013), which is also related to increase in NPP of alpine grassland (Wang et al., 2017). However, the response of alpine grassland to climate warming varies with different moisture gradients and grassland types. In the relatively wetter southeastern Tibetan Plateau, an increasing temperature can promote vegetation growth; however, in the drier northwestern Tibetan Plateau, a higher temperature would limit vegetation growth (Huang et al., 2016; Xu, Chen, & Levy, 2008). Plant growth in alpine meadow is mainly limited by temperature, but limited by both temperature and soil moisture in alpine steppe (Wang Yi, et al., 2016; Wang, Zhang, et al., 2016). In addition, precipitation variation plays a crucial role in the interannual variation of NDVI or NPP for alpine grassland (Ding et al., 2007; Shi et al., 2014). Under the context of universal warming since 2000 (Figure S1), differences in precipitation may have been one of the most significant factor driving the unconformity in NDVI trends between the northeast and southwest Tibetan Plateau (Liu, Shao, & Wang, 2015, 2015).

### 3.2 The impact of human activities on alpine grassland

Human activities play an increasingly important role in the change of alpine grassland, with grazing considered as the main component of human‐induced disturbance (Chen et al., 2014; Yu et al., 2012). Overgrazing is considered as one of the most important driving factor in the early stage of alpine grassland degradation (Gao & Li, 2016), and is the primary cause of grassland degradation in local areas (Bai, Zhang, Xie, & Shen, 2002; Li et al., 2010; Wang et al., 2012; Zhang, Gao, et al., 2015; Zhou et al., 2005).

Manipulative experiments and field observations showed that grazing activities decrease the height and coverage of alpine grassland (Duan et al., 2010). According to the result of meta‐analysis from Lu et al. (2017), grazing could reduce aboveground biomass by 47%. Comparisons between grassland inside (no grazing area) and outside fences (free grazing) have emphasized that fencing enclosure could improve aboveground productivity, and vice versa (Wu, Du, Liu, & Thirgood, 2009; Yan & Lu, 2015; Zhang, Gao, et al., 2015, 2015).In addition, the responses of aboveground biomass to grazing are also influenced by precipitation and elevation (Wang & Wesche, 2016).

The human populations of Qinghai Province and the Tibet Autonomous Region increased rapidly since the 1960s, and grazing pressure grew before the mid‐1990s, subsequently showed an unsteady decline, and decreased since 2004 due to grassland protection policy introduced (Figure S2). Since the 1980s, grassland contract close to villages and intensive grazing caused local grassland degradation, while remote rangeland was given time to recover (Hafner et al., 2012; Zhao & Zhou, 1999). Multiple studies have demonstrated the significant role of accessibility factors in grassland degradation at fine scales (Gao et al., 2013; Li, Li, et al., 2016; Liu et al., 2006; Wei & Qi, 2016). Over the Tibetan Plateau, with human activity intensity quantified based on population density, road network, and settlement locations, Zhao et al. (2015) further confirmed that human activities accelerated alpine grassland degradation, as evidenced by a faster NDVI decreasing in areas with higher human activity intensity.

In addition, while the human activity intensity increased in local areas and might trigger alpine grassland degradation, conservation projects reduced the impact of human activities on nature reserves, resulting in NPP increase and improvement of alpine grassland (Zhang, Hu, et al., 2016). However, in local areas of nature reserves, the existence of negative impacts of human activities is undeniable. For instance, growing population and mineral exploration lead to grassland degradation in the marginal area of the Altun Mountain nature reserve (Liu, Zhao, et al., 2015).

### 3.3 The relative impact of climate change and human activities on alpine grassland changes

Most current studies at a broader spatial scale use statistical models or mechanistic models with remote sensing products, such as the Residuals‐Trend model, to disentangle the relative importance of climate change and human activities on alpine grassland dynamics (Tables 1 and 2).

**Table 1:** Summary of studies focusing on distinguishing the respective contributions of climate change and human activities on alpine grassland variation across the Tibetan Plateau

No.	VI dataset (period)	NPP_P_ method	NPP_A_ method	Main driver		Reference
				Climatic force period (percentage)^a^	Human activities period (area percentage)^a^	
1	GIMMS_2g_–NDVI (1982–2006), MODIS–NDVI (2001–2011)	Terrestrial ecosystem model (TEM)	Carnegie–Ames–Stanford Approach (CASA)	1982–2001 (79.62%); 2001–2011 (56.59%)	1982–2001 (20.16%); 2001–2011 (42.98%)	Chen et al. (2014)
2	GIMMS_3g_–NDVI (1986–2011), MODIS–NDVI (2000–2011)	—	—	1986–2000 (82.3%); 2000–2011 (90.6%)	—	Huang et al. (2016)
3	MODIS–NDVI (2000–2012)	—	—	2000–2013	—	Lehnert et al., (2016)
4	GIMMS–LAI (1982–2009)	—	—	1982–2009	—	Zhu et al. (2016)
5	GIMMS_3g_–NDVI (1982 to 2013)	—	—	1982–2013 (33.93%)	1982–2013 (66.07%)	Pan et al. (2017)
6	MODIS–NDVI (2000–2013)	CASA	CASA	2001–2013 (56.7%) for grassland degradation	2001–2013 (28.6%) for grassland restoration	Wang, Zhang, et al. (2016)
7	MODIS–NDVI (2000–2014)	Thornthwaite Memorial model	CASA	2000–2014 (67.3%) for mitigation of desertification	2000–2014 (58.6%) for exacerbation of desertification	Li, Zhang, et al. (2016)
8	MODIS–NDVI (2000–2012)	(Zhou & Zhang, 1995)	CASA	2000–2004 (41.55%); 2004–2012 (83.75%)	2000–2004 (58.45%); 2004–2012 (16.25%)	Xu et al. (2016)

a Period (area percentage) denotes the period that grassland change was caused by the corresponding primary driving factor and the area percentage of contribution from this factor.

**Table 2:** Summary of studies focusing on distinguishing the contributions of climate change and human activities to the variation of alpine grassland across subregions of the Tibetan Plateau

No.	Study area	VI dataset (Periods)	NPP_A_ method	Main driving factors		Reference
				Climate Factor Period (improvement or degradation)^a^	Human Activities Period (improvement or degradation)^a^	
1	TRHR^+^	GIMMS_2g_–NDVI (1988–2005)	GLO‐PEM	1988–2005 (improvement)	—	Fan et al. (2010)
2	SRYR^+^	GIMMS_2g_–NDVI (1982–2006), MODIS–NDVI (2000–2010)	CASA	1982–2010 (key factor for improvement)	1982–2010 (exacerbation of degradation)	Xu et al. (2017)
3	SRY&YR+	Aerial photography (1969) and TM (1989, 2000, 2007 and 2013)	—	1960s–2010s (key factor for degradation)	1960s–2010s (exacerbation of degradation)	Du et al. (2015)
4	TRHR^+^	GIMMS_2g_–NDVI (1982–2006), MODIS–NDVI (2000‐2012)	CASA	1982–2000 (improvement), 2001–2012(degradation)	1982–2000 (degradation), 2001–2012 (improvement)	Zhang, Zhang, et al., (2016)
5	TRHR^+^	SPOT‐NDVI (1998–2012)	—	—	1998–2004 (degradation), 2005–2012 (improvement),	Cai et al. (2015)

SRYR^+^, source region of the Yellow River; SRY&YR^+^, source regions of the Yangtze and Yellow Rivers; TRHR^+^, Three Rivers Headwaters Region.

a Periods (improvement or degradation) are the periods over which grassland change was caused by the corresponding main driver and the direction of the driver affecting grassland change.

#### 3.3.1 The Tibetan Plateau regional perspective

There are four different views for the contribution of alpine grassland variation across the entire Tibetan Plateau region (Table 1). The first perspective is that leading factor of grassland variation is climate change, which is supported by the logic that climate change influences larger proportional areas of grassland than that of human activities (Chen et al., 2014; Huang et al., 2016). Climate change overridden overgrazing to primarily control grassland dynamics on Tibetan Plateau since 2000 (Lehnert, Wesche, Trachte, Reudenbach, & Bendix, 2016). The results based on multiple global ecosystem models have also shown that climate change has played a major role in increasing vegetation greenness on Tibetan Plateau (Zhu et al., 2016). The second perspective is that grassland variations are primarily influenced by nonclimatic drivers, likewise, evidenced by the result that nonclimatic force affects larger proportional areas than climate change (Pan, Zou, Liu, Wu, & He, 2017).

Based on similar methods, however, the third perspective suggests that both climate change and human activities control vegetation dynamics but have inverse influences (Table 1). For instance, Wang, Zhang, et al. (2016) reported that climate change was the primary factor in alpine grassland degradation, while human activities were the dominant factor in grassland recovery. In contrast, Li, Zhang, et al. (2016) suggested that climate change, especially precipitation, played a key role in mitigating grassland desertification, whereas human activities exacerbated grassland desertification. The fourth perspective is that climate change and human activities alternately dominate grassland dynamics. Xu et al. (2016) found that human activities were the leading factor causing grassland degradation before implementing ecological projects, while climatic force became the leading factor in grassland improvement after implementing ecological projects.

**Figure 2:**
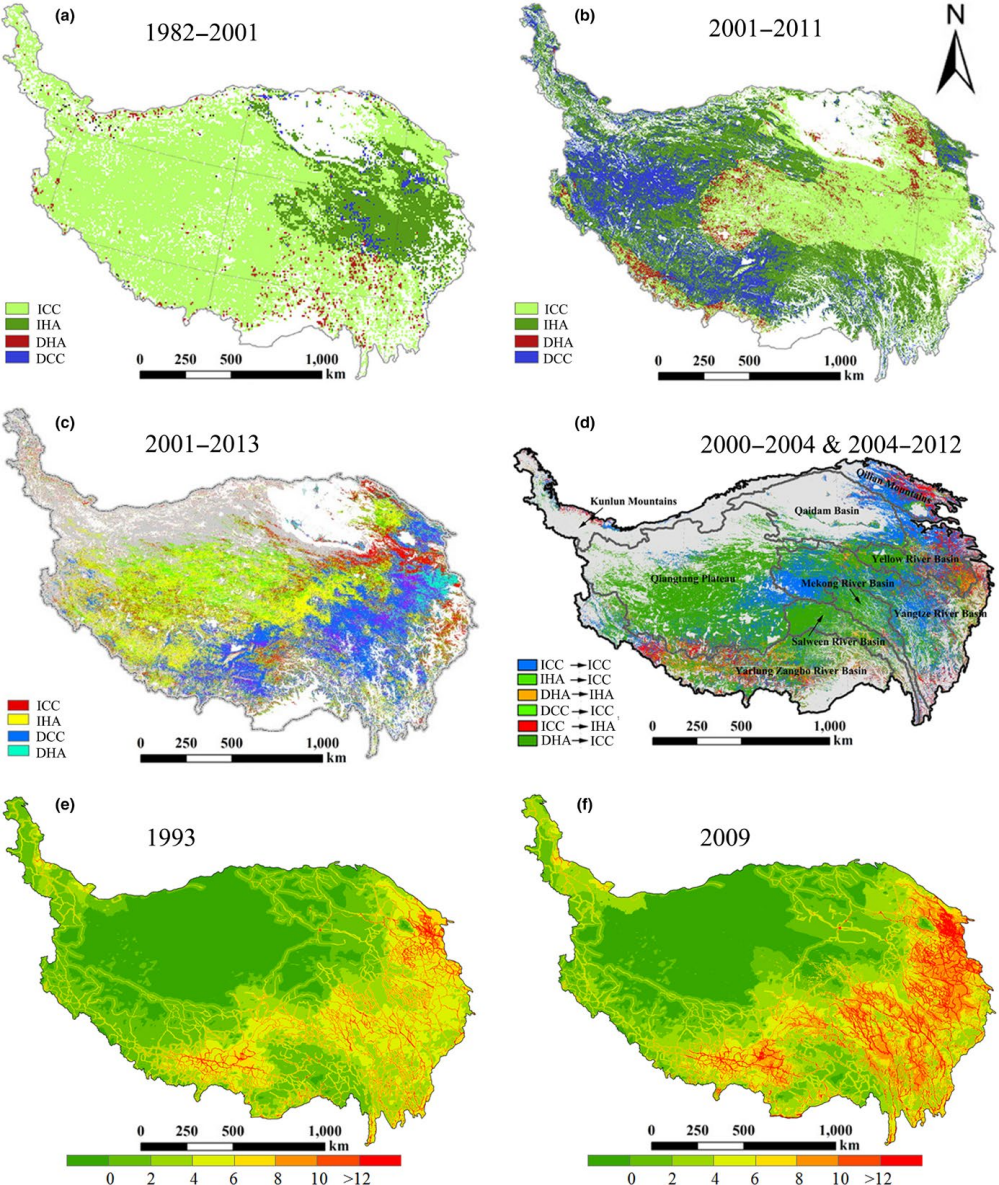
Spatial distribution of attributions for alpine grassland NPP change during (a) 1982–2001 (Chen et al., 2014), (b) 2002–2011 (Chen et al., 2014), (c) 2000–2013 (Wang, Zhang, et al., 2016), and (d) 2000–2004 and 2004–2012 (Xu et al., 2016). Human footprint pressure was mapped in (e) 1993 and (f) 2009 (Venter et al., 2016a, 2016b). In abbreviations in the legends of a‐d panels, *I* indicates an increase in NPP,* D* indicates a decrease in NPP,* C* indicates a change in NPP due to climatic factors, and *H* indicates a change in NPP due to human activities

In the studies mentioned above, the spatial distribution of climate and anthropogenic contribution also showed remarkable differences (Figure 2a–d). Human activity intensity is mainly concentrated in the southern and mid‐eastern Tibetan Plateau, while that is rare in the northwestern Tibetan Plateau, especially in wilderness areas (Figure 2e–f). However, these studies implied that human activities accounted for a large proportion of alpine grassland degradation or restoration in this wilderness areas, although the relative contribution of human activities varied across the different study cases (Figure 2a–d) (Chen et al., 2014; Wang, Zhang, et al., 2016; Xu et al., 2016). Therefore, uncertainties still exist in the relative contributions from climatic and anthropogenic force and require a more reliable estimation.

#### 3.3.2 The Tibetan Plateau subregion perspective

Similarly, researchers have drawn different conclusions in the subregions of Tibetan Plateau (Table 2). For instance, in the Three Rivers Headwaters Region, Fan et al. (2010) and Xu, Wang, and Zhang (2017) suggested that climate change was the key factor for alpine grassland improvement, while Du, Wang, and Li (2015) pointed out that the warm‐dry trend was the leading factor of the degradation pattern of alpine grassland. Some studies have also shown that after implementing ecological engineering, the impact of human activities changed on grasslands transformed from disadvantage to advantage (Cai et al., 2015; Zhang, Zhang, et al., 2016), while the role of climate varied in the opposite directions (Zhang, Zhang, et al., 2016).

## 4 CAUSES OF ATTRIBUTION DIFFERENCES ACROSS THE TIBETAN PLATEAU

Four inconsistent conclusions on Tibetan Plateau and its subregion, including differences in spatial distribution of contribution, were drawn from previous studies using numerous methods. These controversies implied that the complexity of the ecosystem and variations caused by methodological inconsistency (Wang & Wesche, 2016). The factors that produce inconsistencies during analysis include the differences in the source and quality of remote sensing dataset, the structure and parameter of the models, and overlooking the spatiotemporal heterogeneity of human activity intensity.

### 4.1 Differences in remote sensing dataset

The temporal consistency of remote sensing datasets is a prerequisite for obtaining reliable results (Tian et al., 2015). Here, we compared five NDVI time series products (May–September) for alpine grassland across Tibetan Plateau, including NDVI from GIMMS_3g_ and GIMMS_2g_, SPOT, MODIS in Terra (Terra‐MODIS), and Aqua (Aqua‐MODIS; Table S1). These datasets have been widely used to identify attribution of grassland change across Tibetan Plateau in recent studies (Tables 1 and 2).

**Figure 3:**
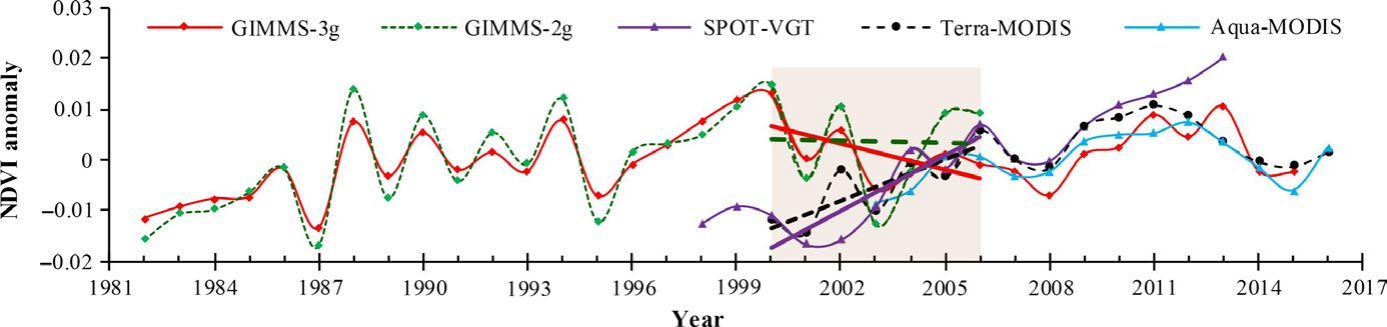
Comparison of the mean growing season (May–September) NDVI on the Tibetan Plateau from different sources. Pixels with growing season NDVI lower than 0.10 are not considered

At the regional scale, positive trend was observed in mean NDVI throughout the growing season since 2000 based on SPOT and MODIS alongside a negative trend in GIMMS_3g_ (Figure 3) (Pang, Wang, & Yang, 2017; Shen et al., 2015). More considerable differences between the NDVI products were detected from 2000 to 2006 than from 2006 to 2012 (Figure 3). During 2000–2006, GIMMS_2g_‐NDVI and GIMMS_3g_‐NDVI showed significant decreasing tendencies, and the latter had a larger descending trend. In contrast, the NDVI derived from Terra‐MODIS and SPOT showed increasing trends during the same period, and SPOT‐VGT had a larger increasing trend. Regarding the spatial patterns of NDVI variation, trends showed large inconsistencies among the Terra‐MODIS, SPOT, and GIMMS_3g_, especially in the western Tibetan Plateau (Shen et al., 2015).

In addition, the quality of remote sensing data derived from the same satellite vary with different time periods or sensors shift. Uncertainties induced by sensors change and degradation can influence NDVI derived from GIMMS and SPOT, especially at the breakpoint due to sensors change (Tian et al., 2015). For instance, Zhang, Zhang, Dong, and Xiao (2013) highlighted that GIMMS_2g_‐NDVI showed abnormal values across the Tibetan Plateau after 2000, which likely resulted in a negative trend of NPP_A_ estimated based on GIMMS_3g_‐NDVI since 2000 (Jiao et al., 2017). To extend the study periods, some researchers used GIMMS_2g_ data with MODIS data to simulate NPP_A_, with taking the year 2000 (Huang et al., 2016; Zhang, Zhang, et al., 2016) or 2001 (Chen et al., 2014) as the time of turning point. Some researchers chose 2004 as the turning point for NPP_A_ simulated using MODIS‐NDVI (Xu et al., 2016) or SPOT‐NDVI (Cai et al., 2015) (Tables 1 and 2). Notably, for Terra‐MODIS, which has thus far not had a sensor change, dataset from the newest updated collection has higher quality than the earlier version datasets, and studies based on earlier version datasets should be reanalyzed (Zhang, Song, Band, Sun, & Li, 2017), but previous studies on Tibetan Plateau have been based on earlier version datasets.

### 4.2 Differences in structure and parameter of models

In most studies described here, the NPP_A_ was derived from CASA model; however, the NPP_P_ can be simulated using numerous methods (Table 1), such as the TEM model (Chen et al., 2014), CASA model (Wang, Zhang, et al., 2016), climate‐driven NPP model (Xu et al., 2016), and Thornthwaite Memorial model (Li, Zhang, et al., 2016). These models are different in parameters and precisions for estimating NPP_P_. Sun et al. (2017) found NPP simulated using climate models was usually two to four times higher than NPP simulated with other estimation models in the Three‐River Headwater Region. Although NPP_A_ simulated with remote sensing parametric models, for example, CASA, have relatively high accuracies, uncertainties remain. Jia et al. (2016) also found that, when estimating grassland biomass across northern China, model parameters may contribute the most to uncertainties, followed by remote sensing data sources and model forms. In addition, while meteorological data are important inputs for model simulations, due to the complicated terrain and insufficient meteorological observations on the Tibetan Plateau, these inputs may also contribute to uncertainties.

Beside, Pan et al. (2016) and Feng et al. (2017) found that the assumptions made in Chen et al. (2014) and Wang, Zhang, et al. (2016) ignored the compensation effect due to grazing. The proportion of grassland productivity consumed by livestock should also be taken into consideration when applying remote sensing‐based models to simulate alpine grassland NPP under the impact of climate change and human activities (Feng et al., 2017). Based on previous research (Chen et al., 2014; Haberl et al., 2014), Pan et al.(2016) used the NPP_gap_ as the deviation in NPP estimation and designed a method to estimate NPP consumption by livestock. The authors then proposed a fixed framework for assessing the impact of climate change and human activities on alpine grassland. Based on this fixed framework, Feng et al. (2017) selected the northern Tibetan Plateau as a case study region to evaluate the variation in NPP_gap_ and its positive or negative value to indicate vegetation dynamics, that is, restoration or degradation.

### 4.3 Neglect of human activity intensity

The intensity of human activity has shown remarkable spatiotemporal heterogeneity across the Tibetan Plateau (Figure 2e–f). However, in the wilderness areas, as mentioned in section 3.3, human activities accounted for a large proportion of grassland degradation or restoration in recent studies (Figure 2a–d). On the contrary, in the areas with high‐intensity human activities, concentrated in the southern and mid‐eastern Tibetan Plateau, there are considerable divergences as well, such as the results in Figure 2a–d. These results could not match with the change of human activity intensity and also not be coincident with the results mentioned in section 3.2 in some local areas.

In addition, regarding the temporal change of human activity intensity, implementation of ecological protection and restoration projects reduced human disturbance in some grassland areas of Tibetan Plateau. However, Xu et al. (2016) concluded that the contribution of human activities on alpine grassland decreased from the period of 2000–2004 to 2004–2012, while Chen et al. (2014) indicated that contribution of human activities showed an increasing tendency from the period of 1982–2001 to 2001–2011. Inexplicably, both of studies ascribed these differences to ecological projects.

## 5 POTENTIAL ROADWAYS TO FACE CURRENT CHALLENGE

Based on sections 3 and 4, we draw a conclusion that there are still some challenges in assessing climatic and anthropogenic contributions to alpine grassland change on Tibetan Plateau. In the future studies, it is important to evaluate the differences among the multiple datasets before inputting some of them into the models and further to improve the accuracy of models by modifying their structure and parameter. Meanwhile, our knowledge of spatiotemporal variations of human activity intensity on Tibetan Plateau is deficient (Li, Zhang, et al., 2017), so this issue should be paid more attention to and be taken into account in the models of attribution assessment for ecosystem change. Particularly, the four views in recent studies mentioned in section 3.3 lacked validation, whereas validating assessments is an indispensable step for quantitatively evaluating the respective attributions of climate change and human activities to alpine grassland variation. Besides, apart from distinguishing the respective attributions, exploring how alpine grassland management interacts with climate change to affect grassland ecosystem (Wu et al., 2017), such as synergistic effects (Dangal, Tian, Lu, Pan, & Pederson, 2016), would be another future research direction.

### 5.1 More attention need on human activity intensity

Emphasizing the impact of human activities on the terrestrial ecosystem is a crucial pathway for promoting the development of physical geography (Fu & Pan, 2016). In recent decades, studies focused on assessing human activity intensity have rapidly developed, including proposing the concept and assessment method for human modification, human footprint, and anthropogenic biomes (Ellis, Klein Goldewijk, Siebert, Lightman, & Ramankutty, 2010; Sanderson et al., 2002; Theobald, 2010). Increasing attention has been paid to evaluating human activity intensity based on related indices, such as population, land use and land cover, and traffic accessibility. Currently, the impact of human activity intensity on ecosystems has attracted increasing levels of research attention, especially for understanding the impact on sensitive ecosystems, including natural reserves (Allan Venter, Maxwell, et al., 2017; Li, Wu, Gong, & Li, 2018; Tapia‐Armijos, Homeier, & Draper Munt, 2017).

Quantifying human activity intensity also becomes an important step to detect the impact of human activities on alpine grassland across Tibetan Plateau in the future studies. It is worth noting that, in the assessment framework mentioned later (section 5.2), a comprehension and quantification of the human activity intensity is of great importance. However, few studies have evaluated human activity intensity across the whole Tibetan Plateau and its subregions (Fan et al., 2015; Li, Zhang, et al., 2017; Zhong, Wang, & Liu, 2008). Furthermore, the data related to human activities have generally been acquired at the certain administrative level, which limits our understanding of the spatial heterogeneity of human activity intensity. Specialization of this data by growing methods, such as simulating the grazing pressure based on household investigations and GPS tracking experiments (Moritz et al., 2010) and simulating the livestock inventory (Nicolas et al., 2016) and population (Gaughan et al., 2016) by a Random Forest regression, could help us better understand the spatial differences in human activities and their impact on alpine grassland.

### 5.2 Framework for validation

The current disagreements regarding the respective impact of climate change and human activities on alpine grassland concentrate on the direction and magnitude of the impacts, which are related to the uncertainties in NPP_P_ and NPP_A_ calculations (Pan et al., 2016). However, most studies based on the Residuals‐Trend model have only validated the NPP_A_, but not the NPP_P_ or the result of attribution (Li, Zhang, et al., 2016; Wang, Zhang, et al., 2016; Xu et al., 2016). Xu, Li, Zhuang, and Pan (2011) suggested that before validating, researchers should choose some samples using a combination of expert knowledge and other auxiliary information. These samples should be located in areas where researchers could explicitly identify the significant attributions of grassland change. Then researchers could validate the assessment by comparing predicted attributions for these samples from models with the previously identified real attributions.

**Figure 4:**
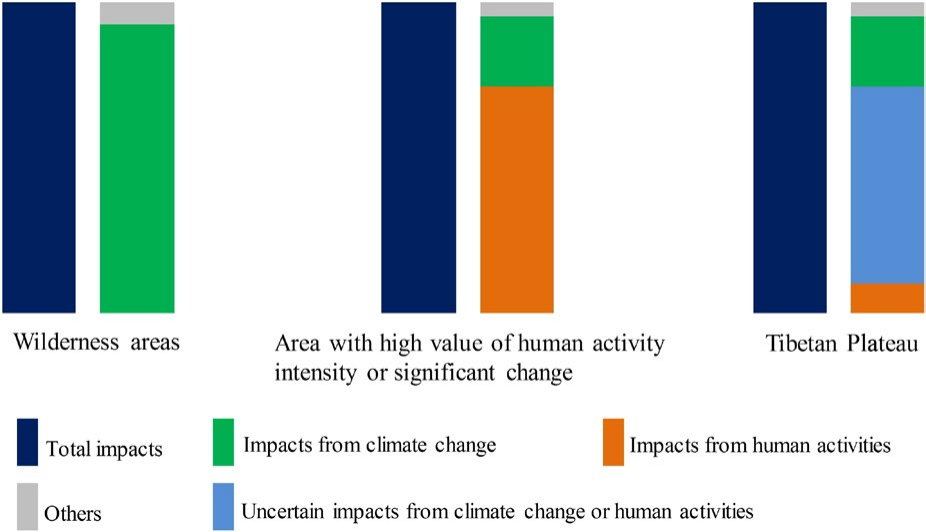
Conceptual graph of climatic and anthropogenic impacts on alpine grassland variation. In wilderness areas, such as northwest Tibetan Plateau, climate change plays a leading role, while in some regions close to roads, settlements, and urban areas, human disturbance determines grassland variation. In addition to human disturbance and climate change, other attributions still exist, for example, the consumptions of wild animals, both in wilderness areas and human‐disturbed areas

**Figure 5:**
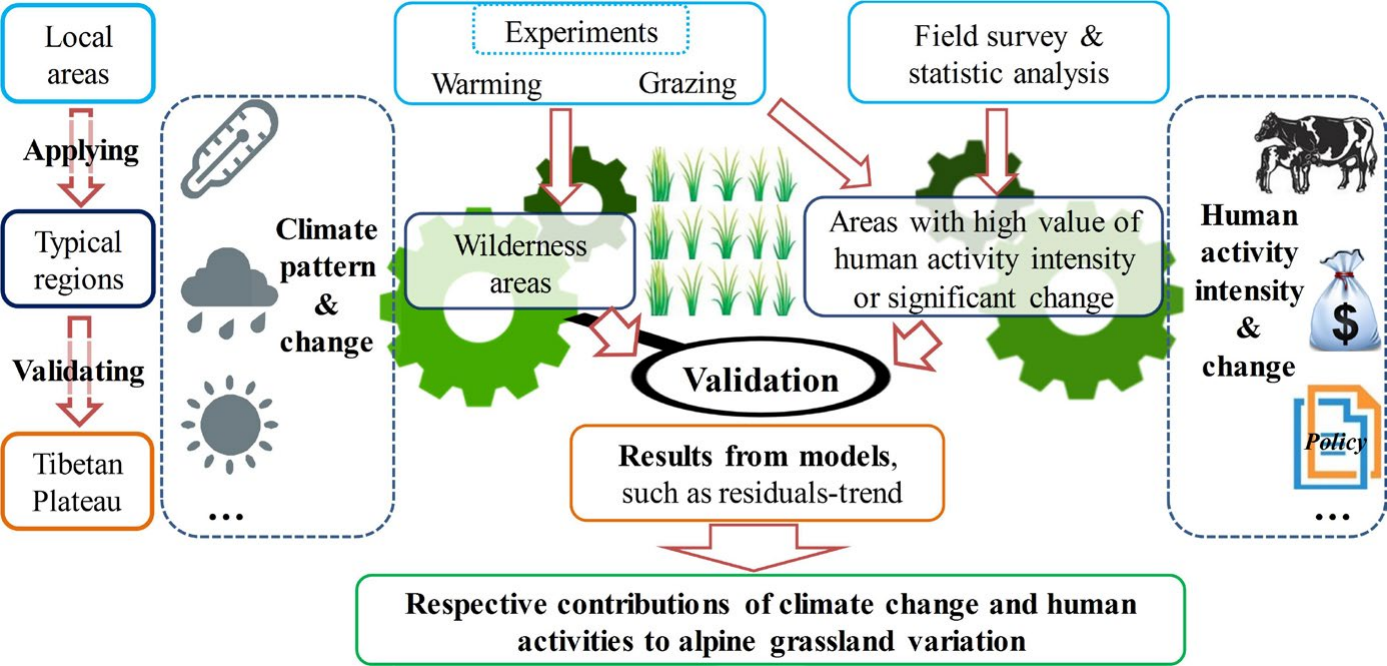
Assessment framework for determining the attributions to alpine grassland change at multiple scales

The wilderness areas in the northwest Tibetan Plateau, which is one of a few wilderness regions worldwide, provide an ideal location to detect climate‐induced alpine grassland variations over a large region (Figure 2e–f) (Allan, Venter, & Watson, 2017; Allan Venter, Maxwell, et al., 2017; Watson et al., 2016). Except for consumption from wild animals, the variations in alpine grassland in these wilderness areas are primarily affected by climate change (Figure 4). In addition, the perennial enclosed areas, where ecological engineering has been conducted since 2005, can also be considered as areas undisturbed by human activities (Chen et al., 2014). Therefore, based on the results of manipulation experiments described in section 3.1, and additional manipulation experiments involving more land cover types and different climate gradients, we could validate the reliability of predicted attributions in the wilderness areas, together with combining the natural features of these areas, for example, patterns of climate change and vegetation characteristics (Figure 5).

Meanwhile, areas with high‐intensity of human activities are concentrated in the southern and mid‐eastern Tibetan Plateau (Figure 2e–f), especially areas near roads, settlements, and urban areas. Thus, areas with high level of human activity intensity or dramatic changes, where grassland variation is mainly affected by human activities, could be selected as typical study areas (Figure 4). Then, based on the study results described in section 3.2 and additional studies in other areas, including manipulative experiment of grazing and buffer zone analysis, the reliability of predicted attribution can be validated (Figure 5).

Finally, through synthesizing the characteristics of wilderness areas and areas with high level of human activity intensity or significant change, we could validate and improve the reliability of attributions across Tibetan Plateau (Figure 5) and then reduce the uncertainties. It should be noted that this framework is also based on existed validation, such as validating the NPP_A_ using field observations.

Furthermore, similar debates on respective contribution of climatic and anthropogenic forces to grassland changes also existed in other regions, such as Mongolia Plateau (Hilker, Natsagdorj, Waring, Lyapustin, & Wang, 2014; Miao, Guo, et al., 2015; Miao, Liu, et al., 2015). This framework for validation might also be applicable to other grassland regions where there are some areas without human activities or natural reserves.

## 6 CONCLUSIONS

As temperature increased significantly and the intensity of human activities grown regionally, no consensus has been reached as to their attributions to alpine grassland variation on Tibetan Plateau. Across the entire Tibetan Plateau and its subregions, four views characterize the recent controversies: the leading driving factor of alpine grassland changes are (1) climatic force, (2) nonclimatic force, (3) combination of anthropogenic and climatic force, or (4) alternation of anthropogenic and climatic force. These views also show spatial inconsistencies and lack validation. The current challenges in distinguishing climatic and anthropogenic contributions to alpine grassland variation on Tibetan Plateau mainly include the uncertainties in quality of remote sensing products, uncertainties in the accuracy of models, especially for simulating the potential vegetation growth, and overlooking spatiotemporal heterogeneity of human activity intensity. Thus, it is necessary to evaluate the differences among the multiple remote sensing datasets before inputting some of them into the models of attribution assessment, to take the spatiotemporal heterogeneity of human activity intensity into account in the models, and further to improve the accuracy of models. We also proposed a framework for accurate validation of anthropogenic and climatic contribution to alpine grassland variation at multiple scales for future studies.

## CONFLICT OF INTEREST

None declared.

## AUTHOR CONTRIBUTIONS

Zhang Y. and Liu L. designed the study, and Li L. wrote most of the first draft. Wu J., Li S., and Zhang H. contributed to the analysis and interpretation of the results. Zhang B., Ding M., Wang Z., and Paudel B. contributed to revise the manuscript and provided conceptual advice. All authors contributed to final approval for publication.
