# Supplementary material for: Current challenges in distinguishing climatic and anthropogenic contributions to alpine grassland variation on the Tibetan Plateau
Source: Ecol Evol. 2018 Apr 27;8(11):5949–63. doi: 10.1002/ece3.4099 (PMC6010758; doi:10.1002/ece3.4099)
Supplement: Supplementary file 1 [file ECE3-8-5949-s001.docx]

**This supplementary file contains two figures and one table, as follows:**


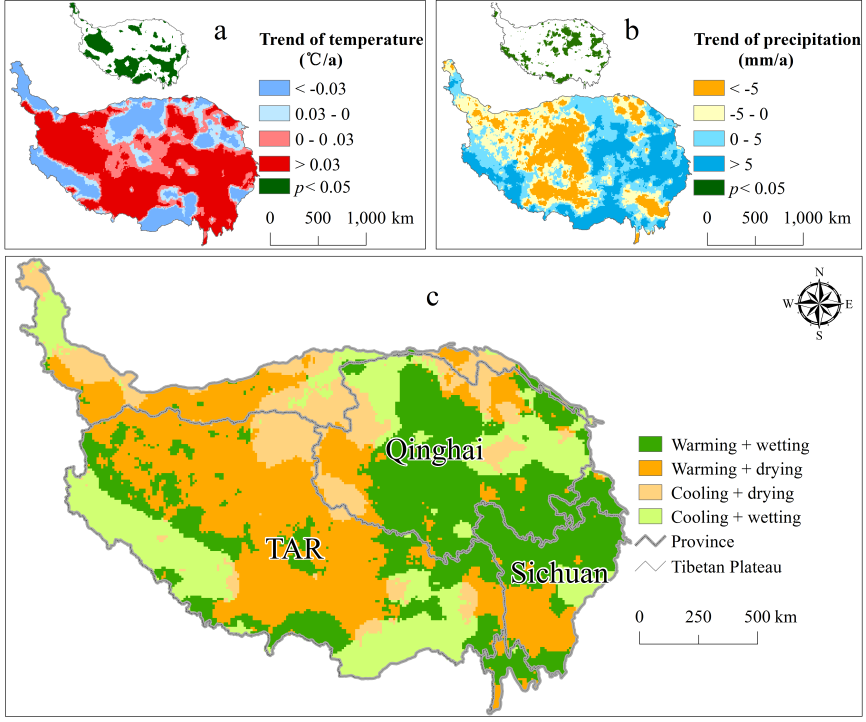


Figure S1 Spatial pattern of temperature and precipitation change during the growing season (May-September) on the TP from 2000 to 2015. Southern Qinghai Province and northwestern Sichuan Province appeared a warm-humid tendency, whereas the mid-northern part of the Tibet Autonomous Region become warmer and drier. Data were obtained from the China Meteorological Forcing Dataset (http://dam.itpcas.ac.cn/) (Chen et al., 2011).

Figure S2 Variation in human population and livestock inventories in Qinghai Province and the Tibet Autonomous Region since 1965. Human populations have increased from 3.62 to 9.12 million over the past six decades. Livestock numbers have grown from 6.39 to 9.53 million from 1965 to the mid-1990s, subsequently showed a shape decline due to the extreme climate, and decreased since 2004 due to a grassland protection policy introduced. Data were obtained from the statistical yearbooks for Qinghai Province and the Tibet Autonomous Region.

**Table S1 Information on the five original NDVI datasets**

| **Dataset** | **Dataset Source** | **Spatial resolution** | **Temporal resolution** | **Period** |
| --- | --- | --- | --- | --- |
| GIMMS_3g_ | https://ecocast.arc.nasa.gov | 1/12°×1/12° | 15 d | 1982–2015 |
| GIMMS_2g_ | http://westdc.geodata.cn | 8 × 8 km | 15 d | 1982–2006 |
| SPOT-VGT | http://www.vito-eodata.be | 1 × 1 km | 10 d | 1998–2013 |
| Terra-MODIS | https://ladsweb.modaps.eosdis.nasa.gov | 1 × 1 km | 16 d | 2000–2016 |
| Aqua-MODIS | https://ladsweb.modaps.eosdis.nasa.gov | 1 × 1 km | 16 d | 2003–2016 |
